# Unilateral biportal endoscopic technique combined with percutaneous transpedicular screw fixation for thoracolumbar burst fractures with neurological symptoms: technical note and preliminary report

**DOI:** 10.1186/s13018-023-04063-2

**Published:** 2023-08-08

**Authors:** Dasheng Tian, Huazhang Zhong, Bin Zhu, Lei Chen, Juehua Jing

**Affiliations:** Department of Orthopaedics and Spine Surgery, The Second Affiliated Hospital of Anhui Medical University, Hefei, 230601 China

**Keywords:** Thoracolumbar fracture, Neurological deficits, Unilateral biportal endoscopic, Endoscopic spine surgery, Percutaneous transpedicular screw

## Abstract

**Background:**

Previous studies on thoracolumbar fractures with neurological symptoms have focused on how to achieve satisfactory fracture reduction, adequate nerve decompression, and stable spinal alignment. With the development of the minimally invasive spine surgery technique, achieving satisfactory treatment results and reducing iatrogenic trauma at the same time has become a new goal of spinal surgery. This research used percutaneous transpedicular screw distraction to partially reduce the fractured vertebrae, followed by completing nerve decompression and reducing residual displacement bone fragments with the assistance of the unilateral biportal endoscopic (UBE) technique to achieve full protection of bone-ligament tissue and obtain good clinical efficacy.

**Methods:**

Guide wires were safely inserted into the fractured vertebra and adjacent upper and lower vertebra under the surveillance of anteroposterior and lateral X-ray fluoroscopy. Transpedicular screws were implanted via guide wires on the side with mild neurological deficits or bone fragment compression (the opposite side of the endoscopic operation). A titanium rod was installed and moderately distracted to reduce the fractured vertebra. Then, under the guidance of the endoscopic view, the laminectomy and ligamentum flavum resection were completed according to the position of the protruding bone fragment into the spinal canal, and the compressed dural sac or nerve root was fully exposed and decompressed. An L-shaped replacer was used to reduce residual bone fragments. The ipsilateral transpedicular screws and rod were installed and adjusted to match the contralateral side. The drainage tube was indwelled, and the incision was closed. The preoperative and postoperative images of the patients were evaluated, and the recovery of neurological symptoms was observed.

**Results:**

Surgery was successfully completed on all six patients, and no intraoperative conversion to open surgery was performed. Postoperative images showed good reduction of the protruding bone fragment and good placement of all screws. At the last follow-up, the neurological symptoms of all patients returned to normal.

**Conclusion:**

The UBE technique combined with percutaneous transpedicular screw fixation in the treatment of thoracolumbar fractures with neurological symptoms can effectively achieve the reduction of displaced bone fragments, improve damaged nerve function, stabilize spinal alignment, and protect the integrity of bone-ligament tissue.

**Supplementary Information:**

The online version contains supplementary material available at 10.1186/s13018-023-04063-2.

## Introduction

With the continuous development of socioeconomics, the incidence of thoracolumbar fractures is increasing year by year, and the proportion of neurological deficit sequelae is also increasing. High mortality and disability rates have caused great economic burdens for families and society [1]. Therefore, treatment options for thoracolumbar fractures have been reconsidered. Relief of spinal nerve compression, improvement of spinal kyphosis and reconstruction of spinal biomechanical stability are the primary treatment goals of these patients, and the effectiveness of traditional surgery has been proven [2]. However, traditional posterior surgery does significant damage to spinal bone-ligament tissues, while anterior surgery has a high risk of injury to vessels and organs [3–5]. It brings new trauma while treating trauma, which is not acceptable to the majority of surgeons and patients. Even the minimally invasive percutaneous or transparaspinal approach with transpedicular screw fixation combined with microscope- or tube-assisted spinal canal decompression cannot completely avoid iatrogenic bone-ligament structural damage. Additionally, the surgical risk is high, and the learning curve is steep [6, 7].

Unilateral biportal endoscopic spine surgery (UBE) is a newer technique that has become popular in recent years. It has been widely used in the treatment of cervical, thoracic, and lumbar degenerative diseases can achieve good therapeutic effects and greatly protect the spinal structure [8–10]. The minimally invasive advantage of UBE seems to be perfectly suitable for the treatment of thoracolumbar fractures, but there are few relevant reports. Since the first application of UBE in the treatment of lumbar disc herniation in 2018, our center has completed more than 1000 UBE surgeries, covering various types of cervical, thoracic, and lumbar degenerative diseases, spinal tuberculosis, spinal intraductal cysts, revision surgery, etc. [11]. We have accumulated significant experience in the treatment of complex intraspinal conditions. In this study, we applied percutaneous transpedicular screw fixation combined with UBE ipsilateral decompression or UBE unilateral laminotomy for bilateral decompression (UBE-ULBD) and displaced bone fragment reduction in the treatment of six patients with thoracolumbar burst fractures accompanied by neurological deficits and achieved excellent results[12].

## Materials and methods

### History and presentation

**Fig. 1:**
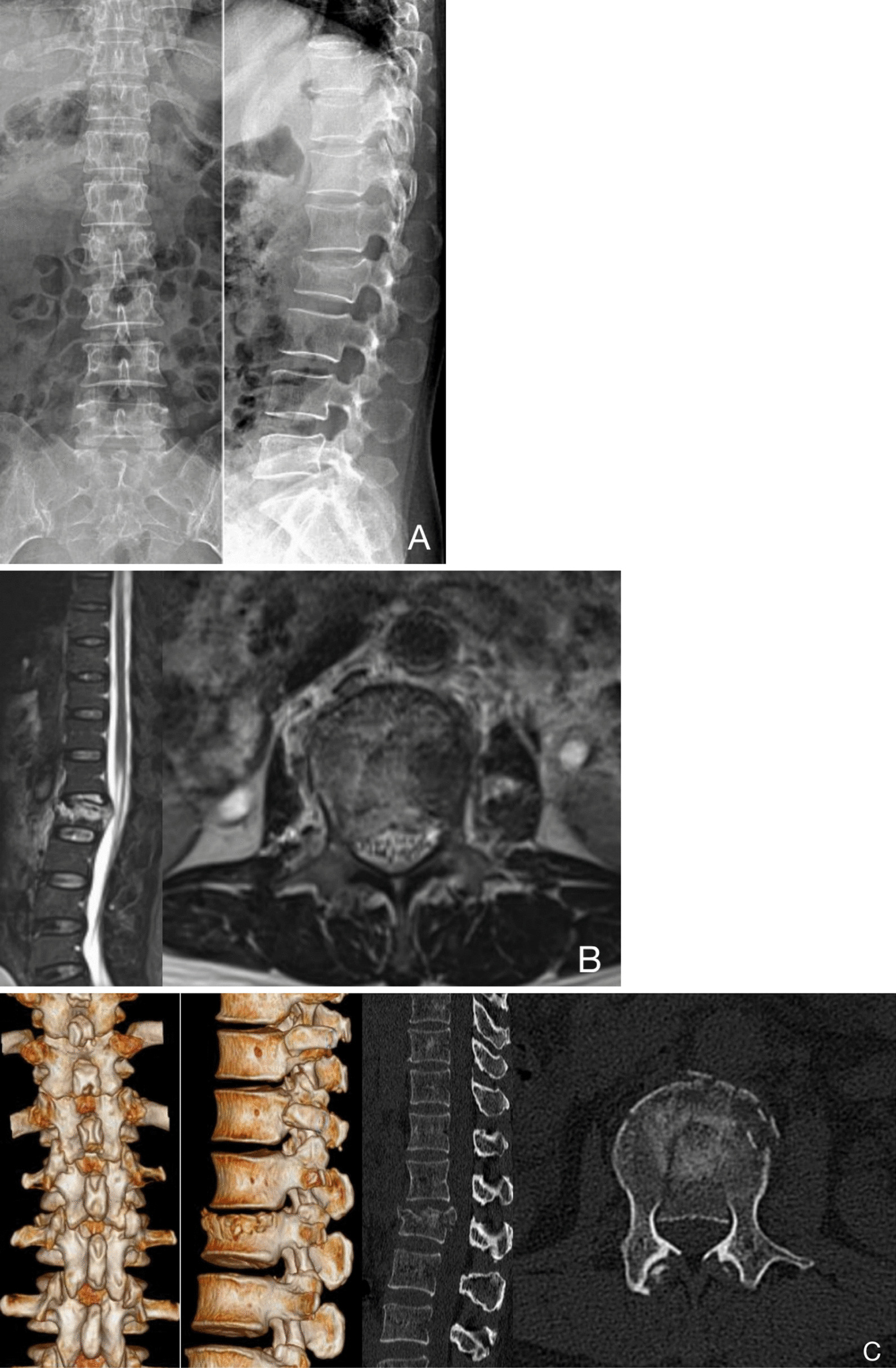
Preoperative images. **A** Radiograph of a 50-year-old patient who sustained a L2 burst fracture after a car accident. **B**, **C** MRI and CT showed that a large bone fragment had herniated into the spinal canal

This study included six consecutive patients with single-level thoracolumbar fracture accompanied by neurological symptoms, including one male and five females, with an average age of 48 (33–71) years. Clinical manifestations were obvious low back pain and unilateral or bilateral lower limb numbness and weakness. The causes of injury included traffic accident injury in three cases, falling injury in two cases, and heavy object injury in one case. Fracture segments included T12 in three cases, L2 in one case, L3 in one case, and L5 in one case. Two patients had a history of hypertension, and two had a history of diabetes. None of them had a history of thoracolumbar trauma or surgery. According to the AO classification of thoracolumbar injury, there were four cases of A3, N2 type and two cases of A4, N2 type [13]. All patients had grade D neurological damage according to the American Spinal Injury Association (ASIA) scale (Table 1). Preoperative radiographic examination, computed tomography (CT) scans and magnetic resonance imaging (MRI) were completed in all patients, which showed different degrees of vertebral burst fracture, kyphosis, posterior vertebral bone fragment invading the spinal canal, and dural sac and/or nerve root compression (Fig. 1).

**Table 1:** Patients’ data

ID	Gender	Age	Fracture level	Fixed level	AO type	ASIA pre-OP	ASIA post-OP (1 week after OP)	ASIA last-FU
1	F	46	T12	T11–L1	A3, N2	D	D	E
2	F	50	L2	L1–L3	A3, N2	D	E	E
3	F	33	T12	T11–L1	A3, N2	D	E	E
4	M	41	L5	L4–S1	A4, N2	D	D	E
5	F	71	T12	T11–L1	A3, N2	D	E	E
6	F	47	L3	L2–L4	A4, N2	D	D	E

### Procedure

#### Anesthesia, position, and instruments

General anesthesia was performed by endotracheal intubation. Invasive circulation monitoring and anesthesia depth monitoring were recommended for elderly patients or patients scoring a high grade on the American Society of Anesthesiologists (ASA) Physical Status Classification System, and controlled blood pressure reduction and satisfactory muscle relaxation were maintained during the operation. The patient was placed prone on the operating bed with the head secured in a headrest. The bilateral upper limbs were placed on the arm plate, and soft pads were placed under the bilateral armpit. Two semi-cylindrical silicone pads were placed horizontally under the shoulder joint and the anterior superior iliac spine, respectively, so that the abdomen was suspended, which facilitated the reduction of the injured vertebra and reduced intraoperative bleeding. Both hips and knees were in flexion.

In the UBE process, we mainly used 30° arthroscopy, a high-speed drill, and radiofrequency, as well as spinal open surgical instruments including strippers, neuroprobes, pituitary forceps, curettes, Kerrison rongeurs, an L-shaped nerve retractor, and an L-shaped replacer.

#### Incision planning

**Fig. 2:**
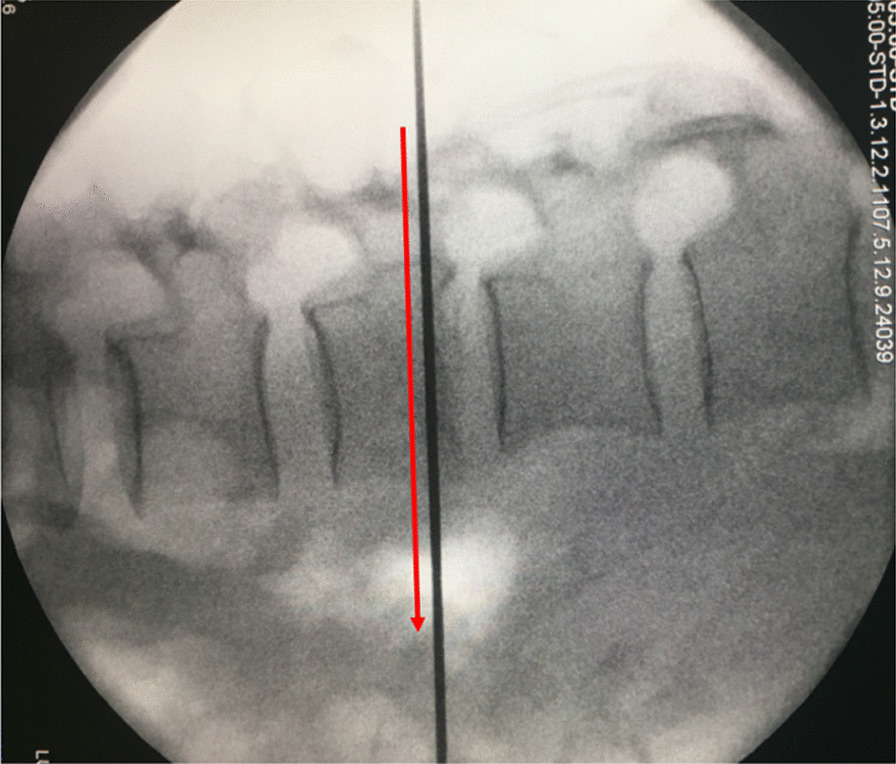
Intraoperative fluoroscopic lateral view shows the fractured vertebra is perpendicular to the ground

**Fig. 3:**
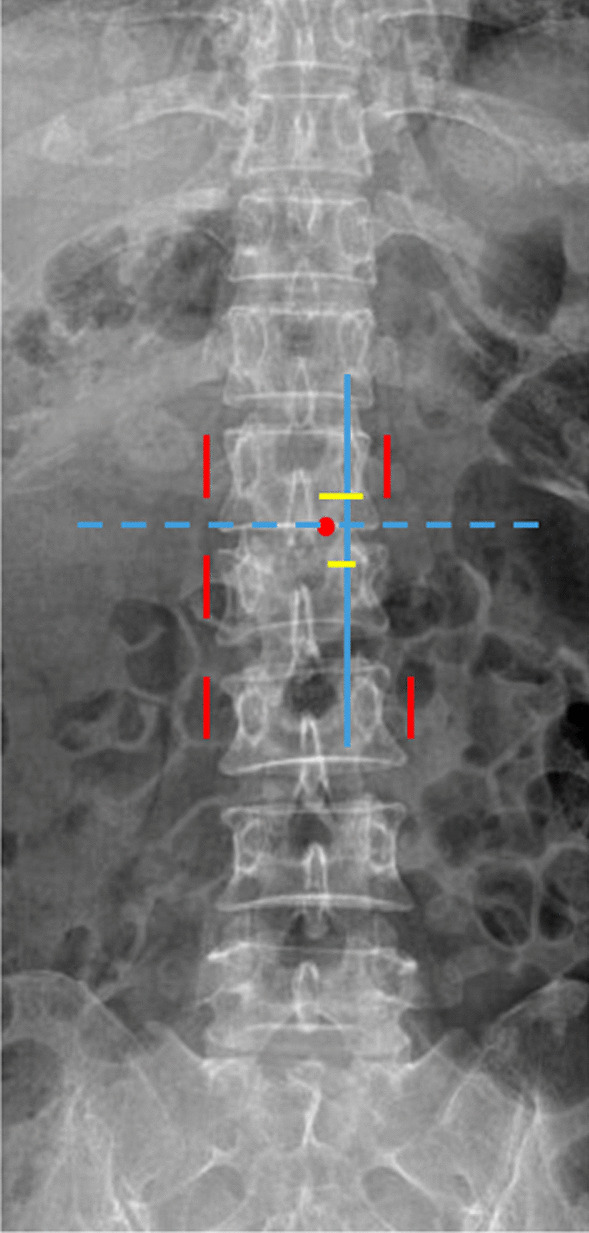
Schematic picture of the lumbar spine. *The solid red lines* represent the incision marks for screws implantation, *the horizontal dotted blue line* is made at the junction of the spinous process and the lamina (*the red dot*), intersecting the inner edge line (*the solid blue line*) of the pedicles, and skin incision marks for two portals (*the yellow lines*) are made 1.5 cm above and below this intersection point

C-arm X-ray was used to obtain standard anteroposterior and lateral images of the spine and confirm the surgical segments. Percutaneous transpedicular screws were placed on the vertebrae superior and inferior to the fracture as well as at the fracture segment on the side with mild neurological symptoms or bone fragment compression. The operating table was adjusted until the fractured vertebra was perpendicular to the ground, and the surface projections of each pedicle were marked (Fig. 2). The side with severe symptoms, or a severe degree of bone fragment compression, was taken as the endoscopic operation side, and the endoscopic operation segment was determined based on this. A horizontal line was made at the junction of the spinous process and the lamina, intersecting the inner edge line of the pedicle, and skin incision marks were made 1.5 cm above and below this intersection point (Fig. 3). The incision length on the left-hand side was about 6 mm, for a viewing portal, and on the right-hand side was about 10 mm, for a working portal (with the right hand as the dominant hand). The size and position of the skin incision could be adjusted appropriately according to different cases.

#### Contralateral percutaneous transpedicular screw reduction

**Fig. 4:**
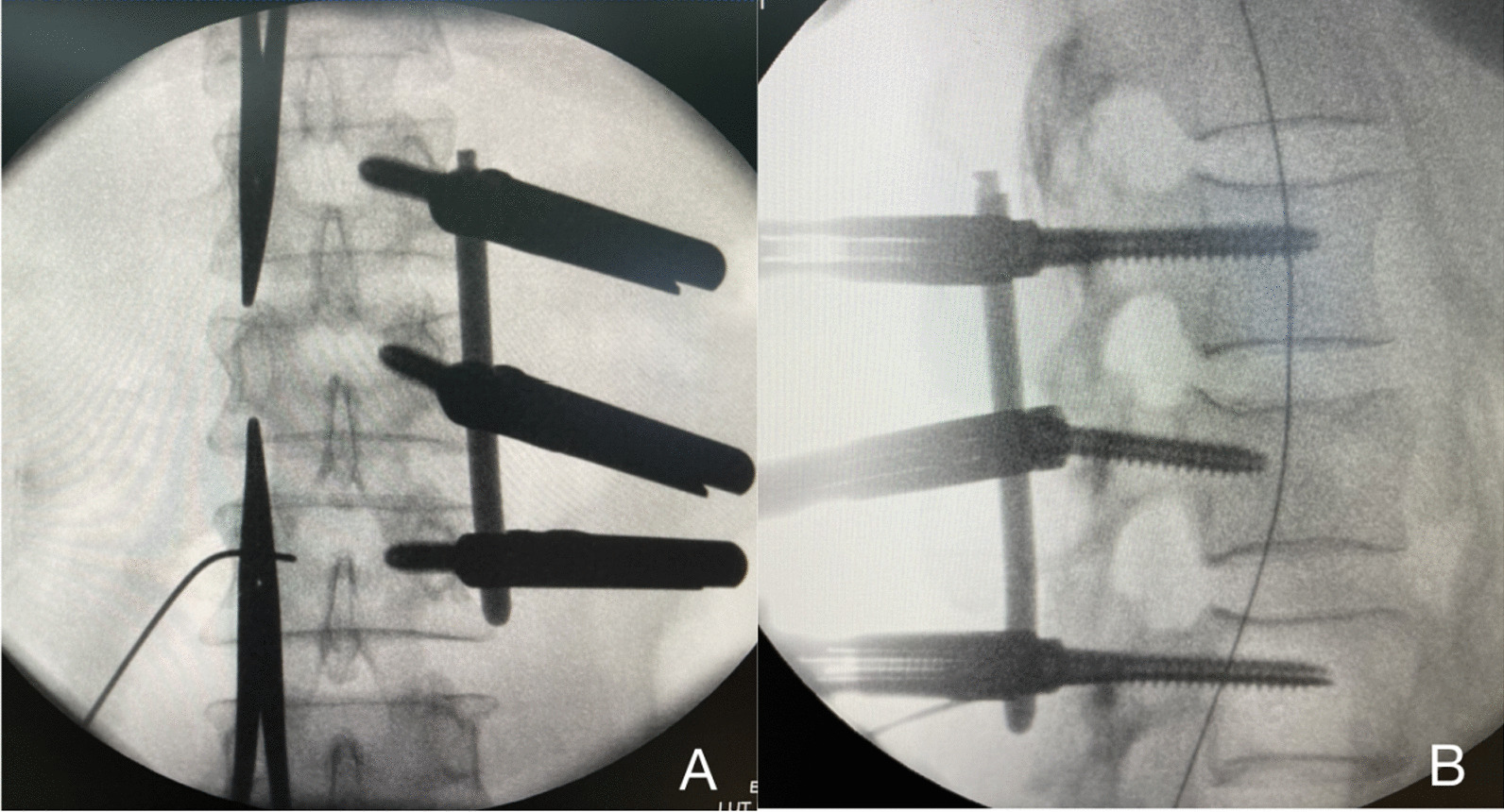
**A**, **B** Anteroposterior and lateral fluoroscopic views show good reduction of the fractured vertebra by the unilateral screw-rod system

Local infiltration anesthesia was performed in the soft tissue route of the transpedicular screw implantation. The fascia was dissected, and the paravertebral muscles were bluntly separated with the fingers to reach the facet joint, in order to reduce postoperative incision pain. Through surveillance of the anteroposterior and lateral images, guide wires were safely inserted from the pedicles. Subsequently, transpedicular screws were implanted through guide wires on the non-endoscopic operation side. The rod was bent according to the degree of vertebral collapse, and the non-endoscopic operation side rod was installed. Next, the posterior wall of the fractured vertebra was distracted, and the anterior height was restored by the unilateral screw-rod system (Fig. 4).

#### Biportal endoscopic neural decompression

**Fig. 5:**
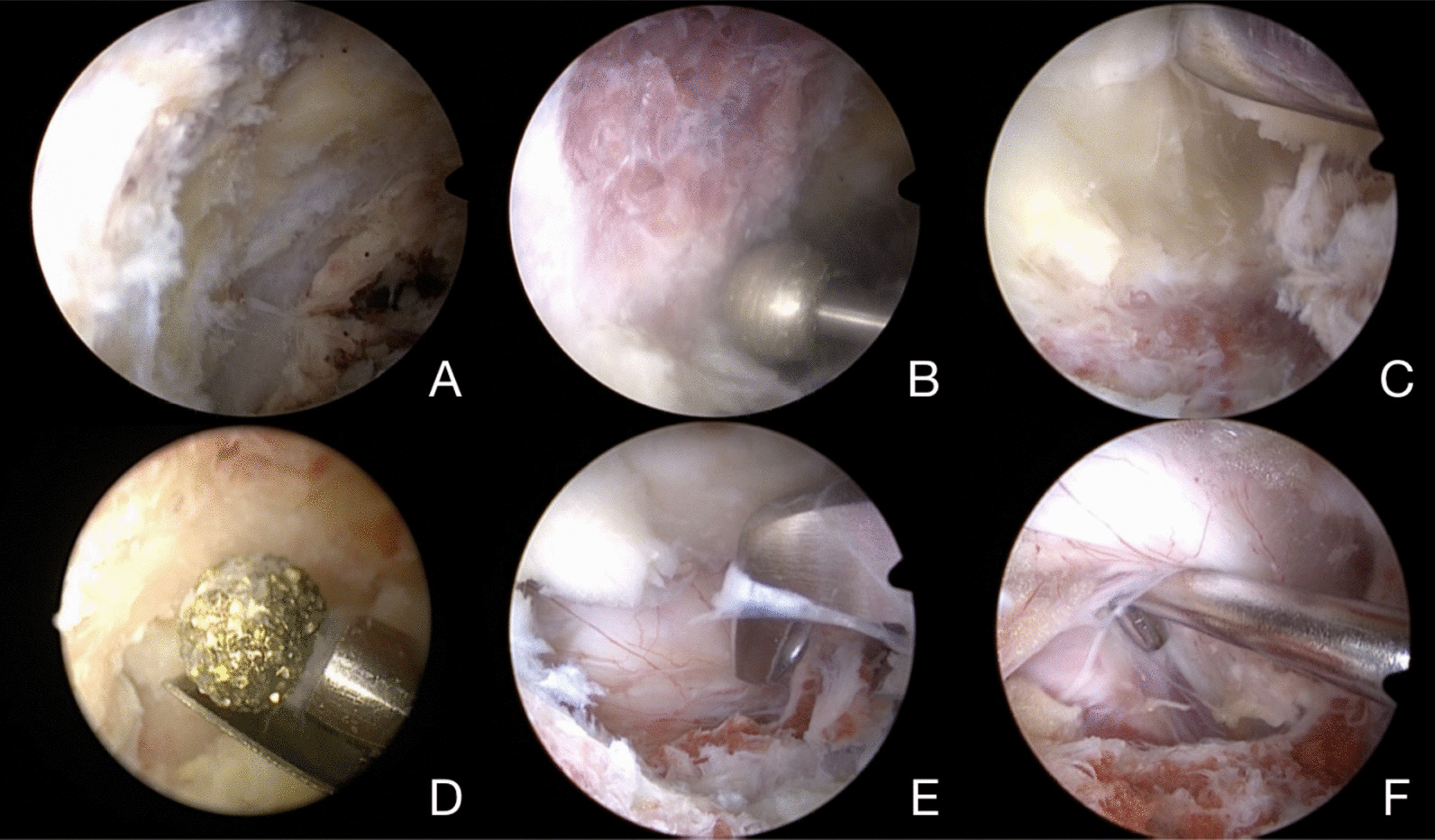
Biportal endoscopic neural decompression. **A** The anatomical structure is clearly exposed under the endoscopic view. **B** Ipsilateral laminotomy is performed with a high-speed drill. **C** The ligamentum flavum attachments are exposed. **D** The spinous process is undercuted by one side with a protective sheath 4-mm diamond bur. **E** The medial facet joint is removed with the Kerrison rongeurs. **F** The retropulsed bone fragment is located below the dural sac

The skin and subcutaneous tissue were cut successively according to the incision marks, and the fascia was cut longitudinally. The primary dilator was passed through the viewing portal route to reach the spinolaminar junction. The soft tissue covered by the lamina surface was bluntly separated by the dilators to form the viewing portal, and the arthroscope with the endoscopic sheath was placed through the viewing portal. Through the incision of the working portal, the dilator was positioned carefully to expand the soft tissue and form the working portal. The dilators used in the cranial and caudal incisions converged at the same point to complete an inflow and outflow saline circuit, which was irrigated by the endoscope. The gravity-perfusate system was connected, and the level of perfusate was maintained at about 50–60 cm higher than the surgical plane. Radiofrequency was applied through the working portal to ablate and coagulate the soft tissue and lead to hemostasis. In this way, anatomical structures such as the lamina, laminar space, ligamentum flavum, and facet joint inner edge can be clearly exposed (Fig. 5A) (Additional file 1).

**Fig. 6:**
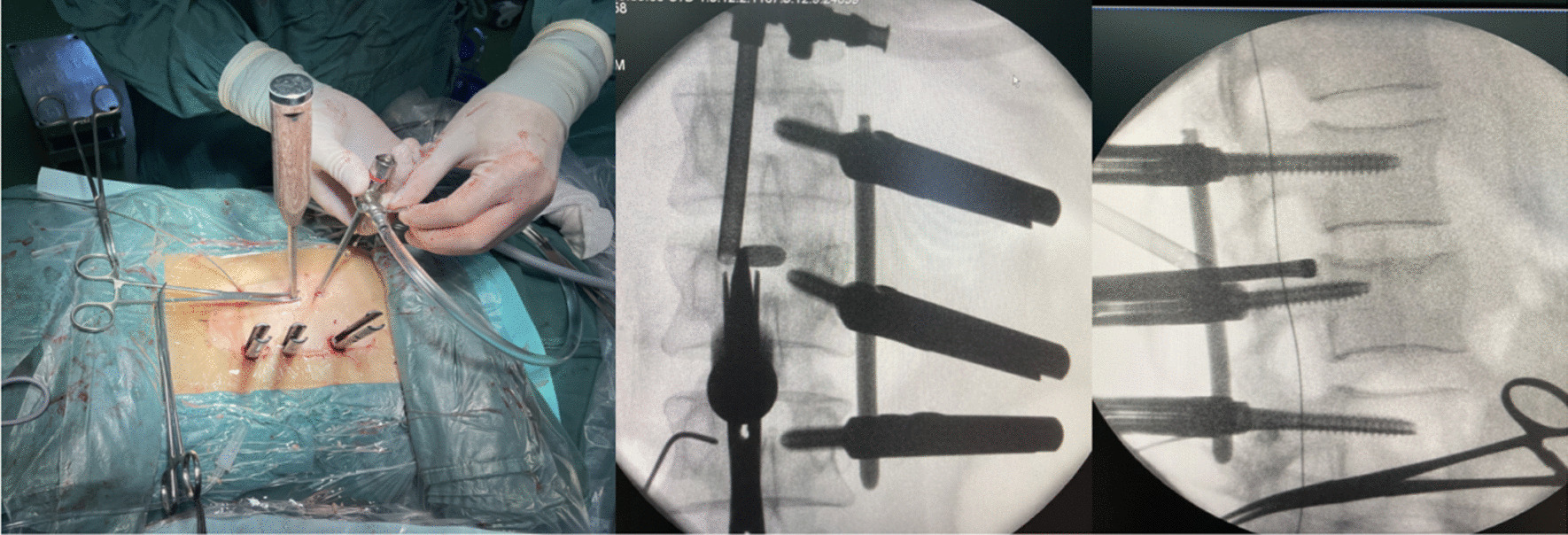
The imaging for the L-shaped replacer in bone reduction process under anteroposterior and lateral fluoroscopic views

The ipsilateral laminectomy was completed with a high-speed drill to expand the laminar space (Fig. 5B). The lamina fenestration area should cover the compression area of the bone fragment in the spinal canal shown by the preoperative images. The ligamentum flavum was removed layer by layer to expose the dural sac and nerve roots (Fig. 5C). If decompression is required on both sides, replace a 4 mm diamond bur, which has one side with protective sheath, to undercut the base of the spinous process (Fig. 5D), dissociate and remove the contralateral ligamentum flavum, grind the deep surface of the contralateral lamina, and expose the contralateral nerve roots. The medial part of the facet joint was removed by high-speed drill or Kerrison rongeurs (Fig. 5E), and the lateral space of the dural sac was expanded to facilitate the entry of the neuroprobe or L-shaped replacer into the spinal canal along the inner edge of the pedicle, and the compression of the bone fragment in the spinal canal was carefully explored (Fig. 5F). The L-shaped replacer was placed between the ventral dural sac and the protruding bone fragment, and then the protruding bone fragment was pushed back into the vertebra (Fig. 6). Small pieces of free bone fragment or ruptured protruding nucleus pulposus should be removed. The neuroprobe repeatedly confirmed the nerve compression, and the bone reduction could be repeated if necessary. The end point of the decompression was reached when the neural elements moved freely in the spinal canal. All operations should be performed accurately and gently to avoid aggravating neurological deficits. After reduction and decompression, complete nerve relaxation was confirmed under endoscopic view and dural sac integrity was checked. If dural sac rupture is found, the perfusate pressure should be appropriately reduced, and the scope of the dural sac tear should be fully exposed. After the herniated nerve is restored, the dura can be sutured endoscopically. If suture is difficult, gelatin sponge or part of fascia can be used to cover the rupture and the endoscopic surgery should be completed quickly to prevent the occurrence of spinal hypertension-like syndrome. The incision should be closely sutured at the end of the operation, and postoperative measures should be taken to reduce the pressure of cerebrospinal fluid to facilitate incision healing.

**Fig. 7:**
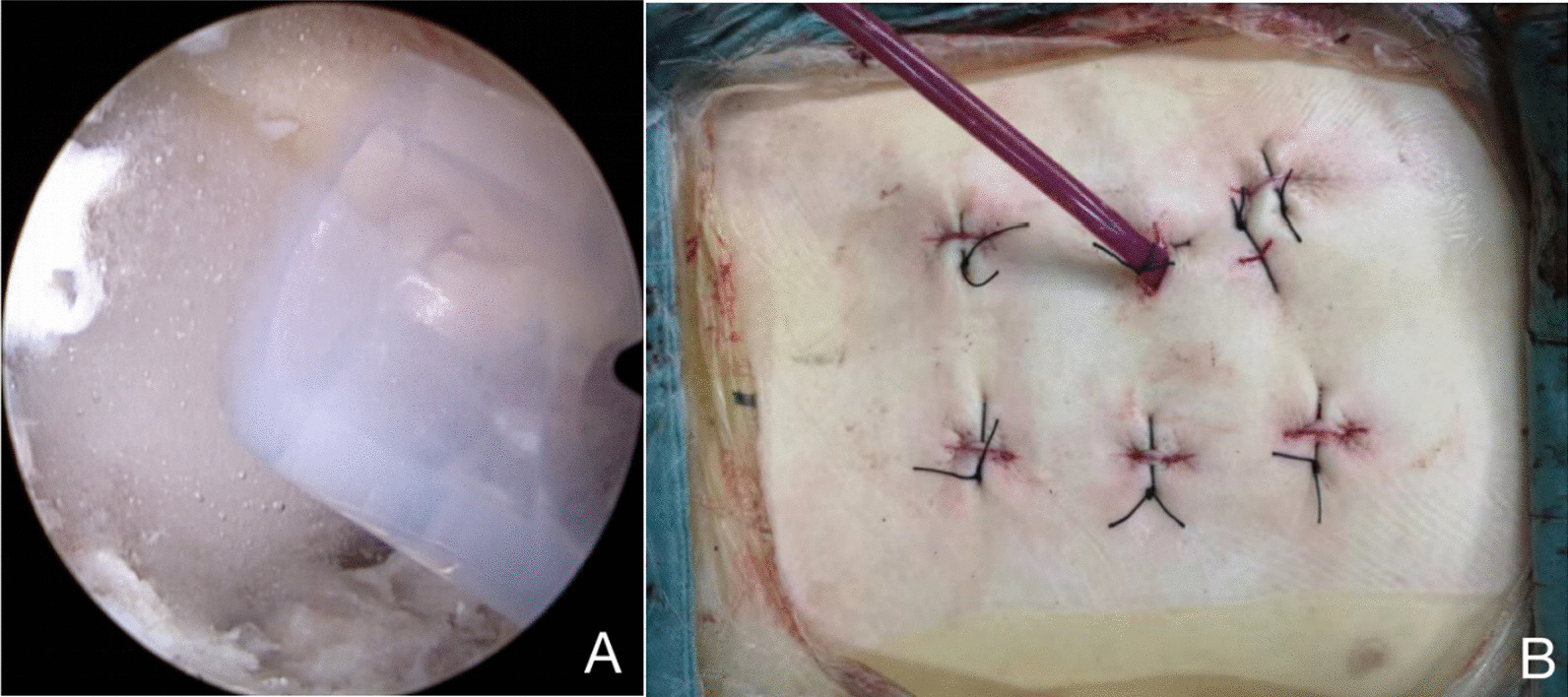
**A** Confirm the position of the drainage tube tip under endoscopic view. **B** Postoperative incision photograph

To evacuate residual fluid from irrigation, or to avoid an epidural hematoma, a drainage tube was placed in the working portal, with its tip placed at the bone-window level, not close to the dural sac or nerve root, to prevent the drainage tip from stimulating the nerve and causing corresponding symptoms after surgery (Fig. 7A). The arthroscope was pulled out slowly to check whether there was active bleeding in the operation field, and hemostasis was fully prevented the occurrence of a postoperative epidural hematoma.

#### Ipsilateral transpedicular screw implantation and incision suture

The remaining transpedicular screws were safely implanted along the guide wires that were inserted ipsilateral to endoscopic operation, and a titanium rod of appropriate length and lordosis was installed and distracted to match the contralateral side. The reduction of the fractured vertebra was completed satisfactorily, and all transpedicular screws were in good position under fluoroscopy. All incisions were washed and tightly sutured layer by layer (Fig. 7B).

### Obvervational index

Visual analogue scale (VAS) was used to evaluate the low back pain preoperation, postoperation (1 week after operation) and at the last follow-up. The sagittal Cobb angle (the angle of the vertical line between the upper endplate of the adjacent superior vertebra and the lower endplate of the adjacent inferior vertebra) and the height of the anterior edge of the fractured vertebra were measured on the lateral X-ray image, and the compression ratio of the height of the anterior edge of the fractured vertebra was calculated: the compression ratio of the height of the anterior edge of the fractured vertebra = (the average height of the anterior edge of the superior and inferior vertebra adjacent to the fractured vertebra − the height of the anterior edge of the fractured vertebra)/the average height of the anterior edge of the adjacent vertebra × 100%.

### Statistical analysis

SPSS 19.0 (SPSS, USA) statistical software package was used for statistical analysis of the data, and the measurement data were expressed as (*X* ± *S*). VAS score, sagittal Cobb angle and the compression ratio of the height of the anterior edge of the fractured vertebra were compared at different time points pre- and postoperation using ANOVA of single factor repeated measurement data, and *q-*test was used for pairwise comparison. The test level *α* value was 0.05 on both sides.

## Results

**Fig. 8:**
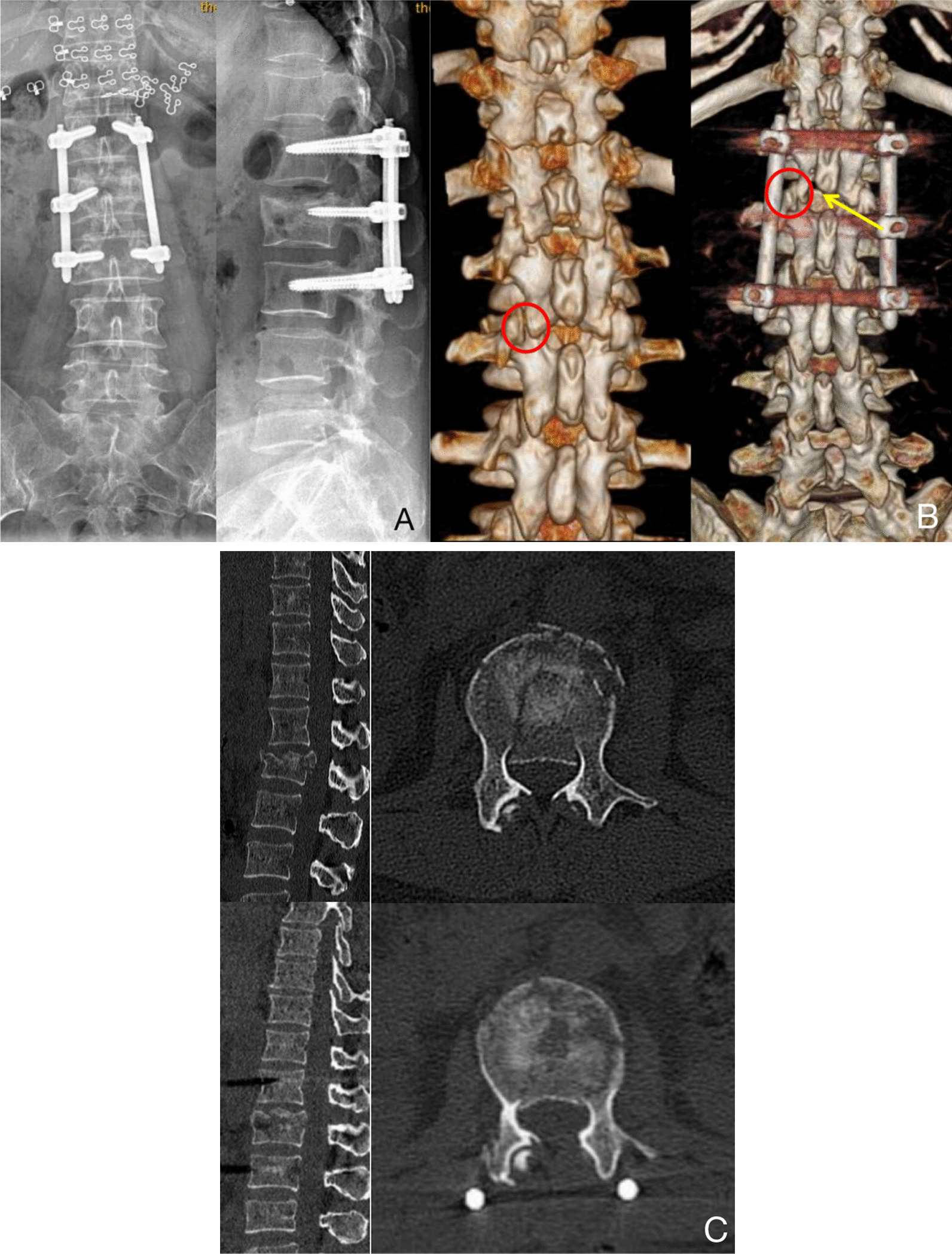
**A** Postoperative radiograph. The fractured vertebra is reduced, and the position of the screw is good. **B** Postoperative 3D images show good lamina fenestration (y*ellow arrow*) and facet joint protection (*red circle*). **C** Lumbar computed tomography scan sagittal and axial views demonstrate the protruding bone is successfully reduced

All six patients successfully completed surgery, and no intraoperative conversion to open surgery was performed. The average surgery time was 110 min (80–140 min) and 39 min (31–50 min) under endoscopy. Antibiotics were routinely used 24 h after operation, and no incision infection occurred. None of the patients required oral or injected analgesics for incisional pain. Functional rehabilitation of lower limbs was started on the first day after surgery to prevent nerve root adhesion. All patients could get up and move under the protection of a rigid brace 2 weeks after surgery. At last follow-up, the symptoms of nerve injury were much improved in all patients, and there was no aggravation of neurological deficits or kyphosis. Immediate postoperative X-ray fluoroscopy showed that the local kyphosis of the fractured vertebra was much improved (Fig. 8A), CT images showed that the protruding bone in the spinal canal was basically reduced, and the transpedicular screws were in good positions (Fig. 8B, C).

The preoperative VAS score was (8.83 ± 1.17) points, which decreased to (3.50 ± 1.05) points postoperation and (1.00 ± 0.63) points at the last follow-up, respectively, which were significantly lower than those preoperation, and the difference was statistically significant (*F* = 133.000, *P* < 0.01, Table 2), indicating that postoperative low back pain was significantly relieved; the difference between the last follow-up and postoperation was statistically significant (*P* < 0.05, Table 2), indicating sustained relief of low back pain during follow-up. The sagittal Cobb angle and the compression ratio of the height of the anterior edge of the fractured vertebra were 18.67° ± 4.63° and 49.90% ± 9.23% preoperation, 2.67° ± 1.86° and 4.98% ± 1.29% postoperation, and 2.83° ± 1.47° and 5.18% ± 1.48% at the last follow-up. All of them were significantly improved compared with those preoperation, and the differences were statistically significant (*F* = 48.157, *P* < 0.01; *F* = 155.461, *P* < 0.01, Table 2), indicating that the height of the fractured vertebra could be significantly restored after surgery and the kyphosis could be improved. There was no significant difference between the last follow-up and postoperation (*P* > 0.05, Table 2), indicating that there was no significant loss of vertebral height during follow-up.

**Table 2:** Obvervational index

Evaluate time	VAS score	Sagittal Cobb angle	Compression ratio
Pre-op	8.83 ± 1.17	18.67 ± 4.63	49.90 ± 9.23
Post-op (1 week after OP)	3.50 ± 1.05	2.67 ± 1.86	4.98 ± 1.29
Last-FU	1.00 ± 0.63	2.83 ± 1.47	5.18 ± 1.48
*F*-value	133.000	48.157	155.461
*P* value	< 0.01	0.001	< 0.01

## Discussion

This study applied the UBE technique combined with percutaneous transpedicular screw internal fixation in the treatment of thoracolumbar fractures with neurological symptoms, which achieved good therapeutic effect and greatly reduced the incidence of approach-related damages, VAS score, sagittal Cobb angle and the compression ratio of the height of the anterior edge of the fractured vertebra were significantly improved after operation. In addition, the advantages of UBE are based on less intraoperative bleeding, faster postoperative recovery, a lower complication rate, and a shorter hospital stay. Two weeks after the operation, all patients were able to get up and move with the protection of a rigid brace. At the last follow-up, the symptoms of nerve injury were significantly improved. Postoperative images showed that the traumatic kyphosis was much improved, the displaced bone fragment in the spinal canal was basically reduced, and the facet joint was very carefully preserved.

Thoracolumbar fractures are often secondary to high energy trauma such as vehicle accidents and falling from heights. Vertebra burst and displacement are often accompanied by tissue injuries such as of nerves, muscles, and ligaments, and in some patients can even be accompanied by chest, abdomen, organ, and brain tissue injuries [1]. The homeostasis of the body is greatly disturbed. For thoracolumbar fractures with spinal cord injury, there is still controversy about the choice of surgical option [5, 6]. Early surgical treatment, maintenance of spinal anatomical sequence and mechanical stability, release of spinal nerve compression and recovery of spinal canal volume, so as to facilitate the recovery of spinal nerve function and reduce postoperative complications, are still the primary goals of treatment for such patients [14]. Traditional surgery requires extensive dissection of paravertebral muscles, and excessive removal of lamina, ligamentum flavum, and other structures to fully expose the intraspinal nerves. Although it can improve nerve function and reduce the protruding bone fragment in the spinal canal, but it is not worth the loss of normal bone-ligament tissues [15].

Unlike open surgery, minimally invasive surgery seems to be more tailored to the treatment needs of traumatic fractures. Microscope- or tube-assisted decompression of the spinal canal and reduction of the vertebra have reduced the approach-related damages, but the limitation of the rigid tube in the air medium makes the operation in the spinal canal impossible to follow one’s will [7, 16]. Since De Antoni et al. [8] first reported the application of two independent portals assisted by arthroscopy in the treatment of lumbar diseases in 1996, and after continuous improvement and promotion by many scholars, the UBE technique, which gradually formed a theoretical system, has been widely used in the treatment of various types of cervical, thoracic, and lumbar degenerative diseases [9, 10]. UBE enables the viewing portal and the working portal to be relatively independent, without rigid tube restrictions, so that the change of the operator’s visual field and the movement of the surgical instruments are more flexible and convenient [17]. In addition, UBE provides a clearer view using water as a medium, which can realize the clearer visualization of anatomic structures, increasing the precision and safety of the surgery [18]. Since the application of UBE in 2021 in our medical center for the treatment of thoracolumbar fractures with neurological symptoms, it has been found that UBE has little impact on severely disturbed homeostasis, and the homeostasis intrusion caused by surgical trauma can be adjusted by the patient’s own recovery ability. In addition, it can maximize the protection of the lamina, facet joint, posterior ligamentous complex (PLC), paravertebral muscles and other structures, and minimize the influence of the surgical approach on spinal sequence balance and biomechanical stability.

Short-segment transpedicular screw fixation has been shown to achieve similar results in kyphosis correction and biomechanical stability maintenance to long-segment transpedicular screw fixation, and the screw placement of the fractured vertebra can better maintain the height of the fractured vertebra [19]. In addition, short-segment fixation can preserve more moving segments and reduce the incidence of adjacent segment degeneration [20]. Generally, the accepted standard treatment for thoracolumbar fractures with neurological deficit is decompression (anterior or posterior) with fusion. However, many scholars still raise objections. Chou et al. [21] believed that short segmental fixation with and without fusion for burst fractures of the thoracolumbar and lumbar spine were comparable, and regional segmental motion could be preserved without fusion, bone graft donor site complications could be eliminated. Juliete et al. [22] also suggested that the use of arthrodesis did not improve clinical outcomes, but it was associated with increased surgical time and higher intraoperative bleeding and did not promote significant improvement in radiological parameters. Similarly, we believe that the reconstruction of the fractured vertebral morphology and the spinal sequence balance is more critical than fusion. Percutaneous transpedicular screw implantation can further reduce the surgical trauma and significantly reduce the probability of injury to the PLC. It is well known that the supraspinous ligament, interspinous ligament, ligamentum flavum, and joint capsule together constitute the PLC. When the load capacity of the anterior and middle column is lost due to thoracolumbar burst fracture, the PLC plays the function of resisting tensile, rotation, shear, and other stresses, protecting the spine and preventing displacement [23]. Once the PLC is damaged, the self-repair ability is poor, and consists mostly of scar repair, so that all biomechanical properties are greatly affected [24]. Percutaneous transparaspinal transpedicular screw implantation can keep away from the PLC completely, and blunt expansion of paravertebral muscles, mainly through dilators rather than radiofrequency sharp dissection, can significantly reduce the damage, and combined with UBE technology it has the advantage of being minimally invasive. This is in line with the trend of minimally invasive spinal surgery.

In order to ensure the successful completion of the operation, some precautions or tips should be paid attention to. First of all, a clean and orderly working environment, tacit and skilled assistant cooperation, safe and appropriate anesthesia state, handy instruments and equipment are all very important to improve the comfort and the efficiency of surgeons. Secondly, regarding the timing of the surgery, many scholars believe that early surgery will aggravate the trauma of the body and significantly increase the amount of overt and latent bleeding, which is detrimental to the already fragile microcirculation [25]. However, combined with our previous experience in open and minimally invasive surgery for thoracolumbar fractures, we believe that, on the premise of ensuring patient safety, early surgery can significantly promote the recovery of neurological function and accelerate the progress of postoperative functional rehabilitation, reduce the incidence of respiratory, circulatory, urinary and other system complications, shorten the postoperative mechanical ventilation time and hospital stay, thereby reducing treatment costs, and the fractured vertebra is easier to reset during the operation, which has more positive clinical significance. Thirdly, for the sequence of screw placement and endoscopic decompression, we believe that the contralateral transpedicular screws should be implanted first, which can distract the collapsed vertebra, facilitate the reduction of the displaced bone fragment in the spinal canal, expand the gap between the dural sac and the displaced bone fragment, facilitate the placing of instruments into this space, and reduce the risk of iatrogenic nerve injury. The guide wire reserved on the ipsilateral side does not interfere with the movement of the endoscope or the operating instruments. Endoscopic neural decompression and bone fragment reduction is the key step of the whole operation. In view of the dural sac obviously being compressed by the displaced bone fragment and the nerve buffering space being narrow, when surgeons choose endoscopic surgery for traumatic thoracolumbar burst fractures, it is a big concern to ensure that the operating instruments enter and leave the spinal canal without disturbing the spinal nerve and aggravating the neurological deficits. In the operation, we found that the contralateral screws can partially reduce the fractured vertebra, and after ipsilateral laminectomy, the base of the spinous process and the deep surface of the contralateral lamina can be further undercut to expand the space in the spinal canal. In addition, for lower lumbar vertebra fractures, L-shaped nerve retractors can be used to enter the spinal canal along the medial wall of the pedicle and pull the dural sac toward the midline to obtain a larger operating space, which is convenient for the L-shaped retractor to reduce the protruding bone. For thoracic or thoracolumbar fractures, because the spinal cord is more susceptible to traction injury and the shape of the spinal canal is different from that of the lower lumbar vertebra, the lateral bony structure of the spinal canal can be appropriately removed, and the lateral space of the dural sac can be expanded to facilitate the operation of instruments. However, it should be noted that the resection of the facet joint should not exceed 1/2. Fourthly, reducing intraoperative bleeding and ensuring a clear endoscopic view are prerequisites for the successful completion of the operation. Appropriate depth of anesthesia, good blood pressure control, and smooth circulation of perfusion fluid are the keys to intraoperative bleeding control. Under the premise of ensuring the perfusion of vital tissues and organs, the ideal controlled hypotension requires the control of intraoperative systolic blood pressure in the range of 90–100 mmHg or the reduction of mean arterial pressure in the range of 60–70 mmHg (70% of the original mean arterial pressure in patients with hypertension). Once bleeding occurs in the visual field, it is necessary to adjust the view to quickly identify the bleeding point, and accurately apply the radiofrequency to hemostasis. Attention should be paid to reducing the power and avoiding the nerve tissue as much as possible when using radiofrequency in the spinal canal. Bone tissue bleeding can use bone wax to seal the bleeding point. Finally, regarding the selection of surgical indications, we believe that type A thoracolumbar fractures with neurological symptoms in AO classification is the best surgical indication.

There are still some limitations in this study. Firstly, for type B and C fractures with injuries to the posterior bone-ligamentous complex (tension band structures), the specific effects of changes in posterior anatomical structures and their adjacent relationship to endoscopic surgical procedures are not yet known. It is necessary to increase the coverage of surgical indications in the later stage to provide more minimally invasive treatment for more complex types of spinal fractures. Secondly, the number of cases in this study is small, and the follow-up time is short. A multicenter large sample prospective randomized controlled study still needs to be carried out to improve the level of evidence. Finally, as we all know, the maintenance of normal sagittal alignment after the surgery in long-term is an important measurement, however, we don't have such data, which needs to be further improved in the future study.

## Conclusion

Due to its advantages of clear visual field, good bleeding control, and flexible and convenient operation under endoscope, this UBE technique can ensure adequate reduction of the displaced bone fragment, and adequate decompression of the injured nerve. Even if bilateral bone fragment compression occurs, the unilateral approach can complete bilateral decompression and achieve global bone mass reduction. Combined with the effect of transpedicular screw distraction, it can further improve traumatic kyphosis, and reconstruct and maintain the sequence stability of the spine. Moreover, with the further improvement of instruments and the accumulation of surgical experience, it is expected that more minimally invasive endoscopic surgery methods will be brought to various types of thoracolumbar fractures.

### Supplementary Information

**Additional file 1.** Video 1, which is the demonstration of the surgical process under the endoscope.

## Data Availability

Data may be made available upon request.
